# Report results: Impact of whole‐body resistance exercise timing on mitigating hyperglycaemia‐induced vascular dysfunction in healthy adults

**DOI:** 10.1113/EP093099

**Published:** 2026-03-09

**Authors:** Laura K. Smith, Lauren Roach, Courtney R. Chang, Tannia Cyriac, Kate Oetsch, Evelyn B. Parr, Monique E. Francois

**Affiliations:** ^1^ School of Medical, Indigenous and Health Sciences, Faculty of Science, Medicine and Health University of Wollongong Wollongong New South Wales Australia; ^2^ Mary MacKillop Institute for Health Research, Faculty of Health Sciences Australian Catholic University Melbourne Victoria Australia

**Keywords:** endothelial function, glycemic control, physical activity, prevention

## Abstract

Postprandial hyperglycaemia is characterized by elevated blood glucose levels following a meal and is associated with impaired vascular endothelial function. Regular exercise has been shown to preserve and improve vascular endothelial function. However, whether there is an optimal time to exercise to mitigate postprandial hyperglycaemia‐related vascular dysfunction is unknown. This randomized crossover study recruited healthy adults to compare four exercise timing conditions with a non‐exercise Control. Participants performed whole‐body resistance exercises at one of 30 min pre (30Pre), immediately post (IP), 30 min post (30Post), or 60 min post (60Post) consuming a high carbohydrate meal. Measures of blood glucose and endothelial function (via flow‐mediated dilation of the brachial artery) were assessed at fasting and incrementally to 120 min following the meal. Exercise performed IP improved flow‐mediated dilation by 2.3 ± 2.7% (*P =* 0.04) and lowered postprandial glucose by 1.5 ± 1.8 mmol/L (*P <* 0.01) at 1 h postprandially, compared to the Control. 30Post and 60post significantly decreased glucose post exercise by 1.06 ± 2.26 mmol/L (*P =* 0.002) compared to the control. Comparatively, pre‐meal exercise did not improve postprandial vascular function or glucose compared to the control or other timing conditions. These findings add precision to the existing literature on postprandial exercise by identifying immediately after eating as most effective for improving vascular and metabolic outcomes relevant to cardiometabolic disease prevention.

## Abstract (highlights)

- **What is the central question of this study?**
  What is the optimal timing for exercise in relation to a meal for mitigating postprandial hyperglycaemia‐related vascular dysfunction?
- **What is the main finding, and what is its importance?**
  Exercising immediately after a meal improved postprandial glucose and endothelial function, whereas exercising before a meal yielded similar glycaemic and vascular responses as not exercising. Exercise is a strategy for mitigating the cardiovascular risks associated with postprandial hyperglycaemia. These findings highlight the importance of exercising soon after a meal to enhance postprandial vascular function and manage postprandial hyperglycaemia.

## 1 INTRODUCTION

Postprandial hyperglycaemia increases oxidative stress, inflammation and endothelial dysfunction (Chang & Yang, 2016; Loader et al., 2015; Meza et al., 2019; Zhu et al., 2007), and has been mechanistically linked with the development of cardiovascular disease (CVD) in individuals with and without type 2 diabetes (T2D) (Katakami, 2018). Efforts to address postprandial hyperglycaemia‐induced vascular dysfunction are still emerging. Exercise and medications that are timed to specifically reduce postprandial hyperglycaemia are effective in mitigating endothelial dysfunction and arterial stiffness under acute controlled conditions (Ceriello et al., 2002; Lunder et al., 2018; Zhu et al., 2007). Exercise lowers postprandial hyperglycaemia and endothelial dysfunction by increasing blood flow and sheer stress, leading to an upregulation of endothelial nitric oxide synthase activity and glucose transporter 4 (GLUT4) translocation (Padilla et al., 2015). Given that the impact of hyperglycaemic ‘peaks’ on vascular dysfunction are more detrimental than constant hyperglycaemia (Ceriello et al., 2008), targeting these peaks by strategically timing exercise around a meal warrants further investigation.

Whole‐body resistance exercise, performed immediately post‐meal, can attenuate the peak postprandial hyperglycaemia and related vascular dysfunction. Previous research has shown that peak glycaemia typically occurs ∼30–45 min post‐meal in otherwise healthy individuals (Bellini et al., 2021; Solomon et al., 2020). Performing exercise immediately after a meal is more effective for lowering postprandial hyperglycaemia than exercising before, or 60 or 90 min post‐meal (Bellini et al., 2021; Solomon et al., 2020), likely attributable to the combined action of insulin and contraction‐mediated glucose uptake (Lai et al., 2010). Notably, postprandial hyperglycaemia disrupts vascular function in a dose–response relationship, with larger postprandial peaks showing greater vascular dysfunction, and is mechanistically considered the most damaging period for vascular function (Ceriello et al., 2008).

The benefits of regular exercise in improving glycaemia management, vascular function and protecting against CVD are well‐known (Fiuza‐Luces et al., 2018; Francois et al., 2018). However, emerging evidence suggests that timing exercise in relation to meal ingestion can optimize cardiometabolic benefits (Bellini et al., 2021; Reynolds & Venn, 2018; Solomon et al., 2020; Zhu et al., 2007). For example, Bellini et al. (2021) and Solomon et al. (2020) showed postprandial glycaemia is reduced to a greater extent when resistance and/or aerobic exercise is performed 0–15 min postprandial, compared to pre‐meal, 60 or 90 min after meal ingestion. In contrast, Reynolds and Venn (2018) found postprandial glucose was reduced more when aerobic exercise was performed 45 min, compared to 15 min, post‐meal. Only one study had investigated postprandial endothelial function; Zhu et al. (2007) found that aerobic exercise (treadmill running) immediately after an oral glucose challenge prevented hyperglycaemia‐induced endothelial dysfunction compared to a non‐exercise control. While these findings indicate that post‐meal exercise is the most effective in lowering postprandial hyperglycaemia, the optimal time to exercise within the post‐meal period to attenuate vascular outcomes requires further research. Further, in the context of vascular function, the impact of exercise timing especially as it pertains to resistance exercise remains to be determined.

Resistance‐based exercise, which engages large muscle groups across the whole body, is an effective modality for lowering postprandial hyperglycaemia (Bellini et al., 2021; Solomon et al., 2020) and has also been shown to improve vascular function (Francois et al., 2016). Importantly, whole‐body resistance exercise is generally well tolerated in the postprandial period, making it a practical and feasible intervention for most individuals (Au et al., 2021). While Au et al. (2021) reported that resistance exercise performed 90 min prior to a meal was ineffective in preventing reductions in blood flow and vascular conductance following acute hyperglycaemia, their study did not examine the effects of post‐meal exercise. Therefore, further research is warranted to explore whether the timing of resistance exercise can modulate vascular responses to a high‐carbohydrate meal.

Endothelial function is reduced on average 2% following a meal, but this effect is heterogeneous (Thom et al., 2016). Health status, meal type (i.e. macronutrient composition, liquid or mixed meal) and sex all impact the endothelial response to varying degrees (Thom et al., 2016). For our investigation we selected a typical breakfast cereal‐based meal, with the carbohydrate content calculated from lean body mass to induce acute hyperglycaemia in individuals without diabetes. The present study aims to test whether performing whole‐body resistance exercise 30 min before, immediately following, or 30 or 60 min after a high carbohydrate meal mitigates endothelial dysfunction, measured by flow‐mediated dilation (FMD). Given the potential for additive effects of contraction and insulin‐mediated glucose uptake, it was hypothesized that resistance exercise around 30 min following the meal would be the most effective condition to mitigate postprandial induced vascular dysfunction. Secondary outcomes include blood pressure, arterial stiffness, and postprandial insulin and glucose responses. These findings will help define the intervention conditions and timing parameters for future randomized controlled trials aimed at preventing T2D and CVD.

## 2 METHODS

### 2.1 Ethical approval

The protocol for this study was approved by the UOW Human Research Ethics Committee (2020/366) and conformed to the standards set by the *Declaration of Helsinki*. The information sheet was provided and the study protocol was discussed prior to all participants providing written informed consent. The study protocol was published retrospectively (Russell et al., 2021).

### 2.2 Study design

This randomized crossover trial compared four different exercise timing conditions to a Control of high carbohydrate meal with no exercise: (i) 30Pre: 30 min of resistance exercises (∼30% of 1 repetition maximum (1RM)), 30 min before a high carbohydrate meal, (ii) IP: 30 min of resistance exercises (∼30% of 1RM), immediately following a high carbohydrate meal, (iii) 30Post: 30 min of resistance exercises, 30 min after a high carbohydrate meal; and (iv) 60Post: 30 min of resistance exercises, 60 min after a high carbohydrate meal. Order of conditions was randomized using the RAND function in Microsoft Excel. Metabolic and vascular function measures were assessed at baseline and for 2 h following the carbohydrate‐based breakfast meal.

### 2.3 Participants

Participants were aged 25–40 years, were minimally or recreationally active (i.e. participating in <300 min of moderate to vigorous exercise per week and not in training or competition for sports) and were otherwise healthy (i.e. absence of chronic disease). Exclusion criteria included any previously diagnosed health conditions (diabetes, kidney, cardiovascular disease); use of diuretics, anti‐inflammatory or anti‐hypertensive drugs; having an unstable weight (weight change ±3 kg); having smoked (in the previous year); pregnancy; or any injury preventing participation in whole‐body resistance exercise. Participants were screened for contraindications to exercise using ‘The Physical Activity Readiness Questionnaire’ (Warburton et al., 2019).

### 2.4 Familiarization and baseline measures

Participants first took part in two familiarization sessions, which were separated by at least 48 h and occurred 1 week before intervention trials.

#### 2.4.1 Familiarization session 1

Participants first underwent a whole‐body dual‐energy X‐ray absorptiometry (DXA) scan (∼5 min), measuring the total and regional fat and fat‐free mass using a MedixDR (Medilink, Saint‐Quentin‐Fallavier, France) DXA system, to characterize participants’ body composition and calculate relative carbohydrate provisions for the meal challenge. A 1RM test was then performed for each exercise (squats, lunges, leg extension, lat pull down, chest press and shoulder press/bicep curl) using standardized methods (Au et al., 2021; Marlow et al., 2014), to calculate workloads (30% of 1RM) for all the resistance exercise sessions.

### 2.5 1RM test protocol (Marlow et al., 2014)

The participants performed one exercise‐specific warm‐up at a self‐selected light‐intensity workload (10–12 repetitions). 1RM was then determined within four sets, with 3–5 min rest between sets. An initial weight of ∼50–70% of the participant's perceived capacity was used, with resistance progressively increased by ∼10–20% until true 1RM was achieved. Each repetition was performed at the same speed and through the same range of movement. This test protocol is repeated for each exercise (resistance protocol). The final weight lifted was used to determine 30% of 1RM for each resistance exercise during experimental trials.

#### 2.5.1 Familiarization 2

To accustom participants to resistance exercise and ensure correct technique, a 30‐min resistance exercise session at 30% of 1RM for each exercise was conducted by an accredited exercise physiologist (A.E.P.).

### 2.6 Resistance exercise protocol

**FIGURE 1:**
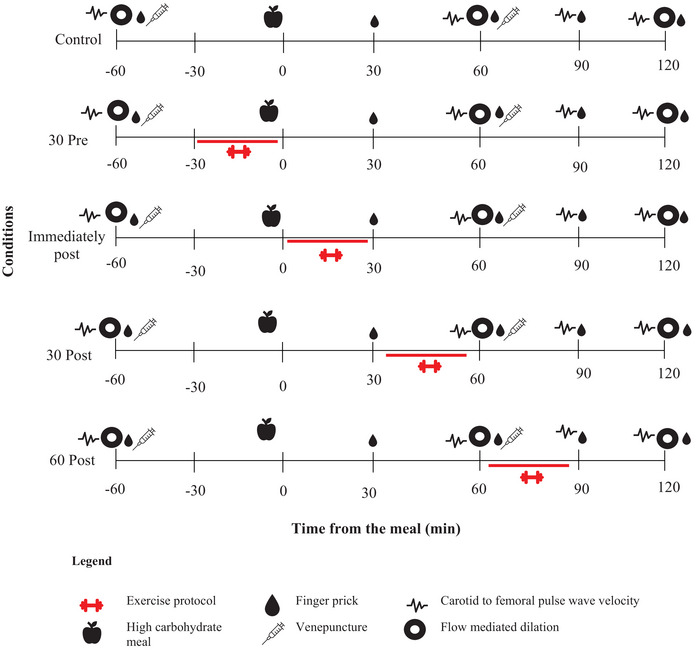
Overview of the study protocol. Each participant completed all five conditions in a random order. Participants arrived having fasted for 10 h, and the high carbohydrate meal was given following baseline measures ∼1 h after arrival. Each measure was conducted at the same time following the meal for each condition.

Participants performed 30 min of resistance exercise for each exercise condition at the predetermined time (Figure 1). Exercises include squats, lunges, leg extension, lat pull down, chest press and shoulder press/bicep curl. These exercises were chosen because they use large muscle groups commonly involved in the usual activities of daily living. Exercises alternated between the lower and upper body to minimize fatigue of a single muscle group. Three sets of 25 repetitions at ∼30% of 1RM, with a ∼1‐min rest between exercises, were completed. Participants completed 5 min of walking at 5 km/h on a treadmill at the start/end of each training session (warm‐up/cooldown).

### 2.7 Experimental conditions

Participants completed the following measures in the same predetermined order for all exercise conditions and Control (Figure 1). Assessments were simultaneously relative to the consumption of the meal (time 0).

#### 2.7.1 Meal consumption

A standard breakfast was used for our proof‐of‐concept. All participants consumed a high‐carbohydrate breakfast meal, within 5–15 min. The standardized meal provided 1.5 g of carbohydrate per kg of lean body mass (LBM). Participants were given 1½ cups of cornflakes and ¾ cup partially skimmed milk, then topped up with a teaspoon (tsp) of sugar and/or juice to make up the total carbohydrates. For example, a 30‐year‐old female with an LBM of 37 kg would have 55.5 g of carbohydrates as 1½ cup cornflakes, ¾ cup skim milk and 2.5 tsp of table sugar.

#### 2.7.2 Blood analyses

Participants arrived at the laboratory at the same time of day for each condition after a ∼10‐h fast. A venous blood sample was collected in a 10 mL EDTA tube to measure insulin in the fasted state and then again at +60 min post‐meal. All samples were centrifuged, and the plasma was aliquoted and stored at −80°C until analysed. Additionally, capillary glucose was collected using a standard lancing device and measured on a HemoCue glucose analyser (HemoCue Glucose 201 RT glucose; HemoCue AB, Angelholm, Sweden) at fasting and 30‐min intervals post‐meal. A Cobas b 101 system (Roche Diagnostics, Rotkreuz, Switzerland) measured lipids (total cholesterol, low‐density lipoprotein cholesterol, high‐density lipoprotein cholesterol, triglycerides) at fasting and +60 min.

#### 2.7.3 Vascular assessments

Vascular assessments of FMD, blood pressure (BP) and arterial stiffness (pulse wave velocity (PWV) and pulse wave analysis (PWA)) were performed at fasting and at 30‐min (pulse wave, BP) or 60‐min (FMD) intervals for 2 h post‐meal (Figure 1). All standardization measures for these assessments were done according to the current guidelines (Laurent et al., 2006; Thijssen et al., 2019).

##### Flow‐mediated dilation

Endothelial function was measured using FMD following the standard guidelines (Thijssen et al., 2019) by a trained researcher (M.F.). FMD of the brachial artery was assessed using a uSmart3300 Ultrasound system (Terason, Burlington, MA, USA) in combination with a semi‐automated analysis system (FMD Studio, Quipu, Pisa, Italy). In preparation for occlusion, a blood pressure (BP) cuff was placed around the forearm (distal to the imaged artery). The brachial artery was imaged longitudinally 2–3 cm proximal to the antecubital fossa. Baseline diameter was recorded for 60 s, after which the blood pressure cuff was inflated for 5 min to >50 mmHg higher than resting systolic BP. After 5 min, the BP cuff was rapidly deflated, resulting in reactive hyperaemia (excess blood in the artery). Post‐deflation diameter was then recorded for a period of 3 min.

##### Brachial artery diameter and blood flow analyses

Edge detection software (Cardiovascular Suite, Quipu) was used to reduce user bias and for continuous simultaneous analysis of brachial artery diameter and blood velocity measures. The FMD response is reported as absolute (mm) and relative (%) change in diameter from baseline compared to peak diastolic diameter following hyperaemia, and to adjust for the potential confounder of baseline diameter (*D*_base_) allometric scaling was used (*D*_base_‐adjusted FMD) (Atkinson & Batterham, 2013). Using a non‐invasive Doppler, blood flow (mL/min) was calculated by measuring the cross‐sectional area of the artery diameter and blood velocity (4 × velocity/diameter). Shear rate (s^−1^) was calculated from both diameter and velocity recordings. The shear rate area under the curve (SRAUC) was calculated automatically from the synchronous diameter and velocity data from cuff release to peak diameter. Baseline and post‐occlusion antegrade and retrograde shear rates were calculated from antegrade and retrograde mean blood velocities (4 × mean baseline antegrade or retrograde velocity/mean baseline diameter) (Atkinson & Batterham, 2013). The ratio of mean blood flow to mean arterial pressure was used to calculate vascular conductance (mL/min/mmHg). The coefficients of brachial artery diameter variation and %FMD are 2.1% and 7.3%, respectively, for the trained researcher (M.F.).

##### Arterial stiffness

Central arterial stiffness was measured using pulse wave velocity with a tonometer (which measures the pulse) on the carotid pulse. At the same time, a cuff placed around the leg is inflated using the SphygmoCor XCEL automated system (AtCor Medical, West Ryde, NSW, Australia). Both pulse transition time (the time for the pulse to travel from the carotid artery to the femoral artery) and the distance between the carotid and femoral arteries were measured. PWV was calculated by dividing the distance between the arteries by the pulse transition time. Pulse wave analysis (PWA) was also measured using the SphygmoCor Pulse Wave Analysis System (AtCor Medical, NSW, Australia). Blood pressure is collected using the PWA automated cuff system around the upper arm. A validated generalized transfer function built into the SphygmoCor software package calculates central aortic pressure waveform, central systolic, diastolic and pulse pressures. Aortic augmented pressure (AP) and the aortic Augmentation Index (AI) were measured. AP is the difference between the uppermost systolic peak on the aortic pulse wave and the pressure at the start of the reflected wave (Russo et al., 2011). The AI is expressed as a percentage and is calculated as a ratio between the AP and the central pulse pressure (PP) (Russo et al., 2011). A built‐in algorithm determined captured signal accuracy for all recordings. In our initial protocol we proposed collecting additional PWV/PWA and BP at 90 min post‐meal. However, logistical constraints prevented the accurate capture of this data at 90 min (with the exercise session in the 60Post condition) and thus vascular measures at 90 min are not reported.

### 2.8 Standardization

To reduce the impact of muscular fatigue and allow for strength reproducibility (consistency in achieving 30% or 1RM during sessions), a break of 48–72 h (Monteiro et al., 2008) between the familiarization sessions and between trial conditions was employed. To minimize the impact of diurnal variation and prior meal consumption, trial conditions began at the same time of the day, in a fasted state, and participants were instructed to eat the same meal the night before each condition.

During vascular measures, participants rested (supine) for 20 min before measurements were taken and refrained from talking or moving during the test (Thijssen et al., 2019). The procedure was conducted at the same time of the day for each trial condition. Participants fasted for approximately 10 h prior to each visit, which included abstaining from caffeine. In the 24 h preceding the visit, they were instructed to avoid alcohol consumption and vigorous physical activity, and to maintain their regular sleep–wake cycle. The same blinded researcher conducted FMD measures for each participant at three points (Figure 1) and for each condition and analysed the resulting images to prevent inter‐rater error. All blood samples were batch‐analysed for insulin in the same run by a condition‐blinded researcher using the Access 2 Immunoassay System, an automated immunoassay system (Beckman Coulter, Brea, CA, USA).

### 2.9 Statistical analysis

Twenty‐seven male and female participants were proposed for this study. The sample size was based on the difference in %FMD and variance at 2 h (Cohen's *d* 0.57) with resistance exercise in adults with type 2 diabetes compared to Control using G*power (α = 0.8) (Francois et al., 2016). Descriptive statistics (means, SD and frequencies) were calculated using SPSS Statistics (Version 28.0.0 (190), IBM Corp., Armonk, NY, USA). Histograms were used to identify outliers using the ±3 SD plot, and the Shapiro–Wilk test was used to test for normality. Data with skewed distributions were considered for log‐transformation before statistical testing (however this was not required). Area under the curve (AUC) was calculated for glucose and FMD using the trapezoid rule, and peak was determined by the max value for measurement. A repeated measures ANOVA was used to analyse the time and condition interaction between conditions in SPSS Statistics (Version 28.0.0 (190)). No sex × condition effect was observed for FMD or glucose. Therefore, sex was not retained as a factor in the final model. Significance was set as *P <* 0.05. Where a significant time and condition interaction was present, and a Tukey *post hoc* analysis was used to compare conditions relative to Control, and between conditions at each time point.

## 3 RESULTS

One hundred thirty‐four people responded to the recruitment advertising. After phone screening, thirty‐one participants were recruited and randomized. Of these, twenty‐four participants completed the full crossover study (M:F, 12:12) (see Supporting information Figure S1). Five participants were excluded from the FMD results due to poor video quality, leaving *n* = 19 in the FMD results (M:F, 10:9).

### 3.1 Postprandial flow mediated dilation

**FIGURE 2:**
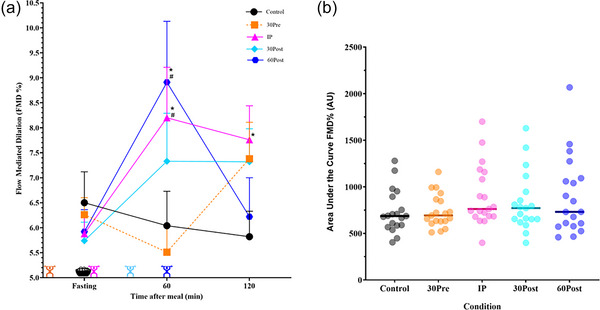
(a) Fasting flow‐mediated dilation (FMD) followed by Control, or exercise 30Pre, IP, 30Post and 60Post meal with FMD measured at 60 and 120 min post‐meal (*n* = 19). 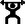 indicates time exercise occurred; 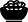 indicates when the meal was eaten. Time point *P < 0.05 compared to control, #*P <* 0.05 compared to pre meal exercise. (b) Area under the curve (arbitrary units (AU)) for postprandial flow‐mediated dilation with exercise 30 min pre‐, immediately post‐, 30 min post‐, and 60 min post‐meal, compared with no exercise control (*n* = 19).

A time‐by‐condition interaction was observed for postprandial endothelial function (%FMD, *P =* 0.00348, Figure 2a, Table 1, Supporting information Table S1). Compared to the control at 60 min post‐meal, FMD was +2.3 ± 2.7% higher for IP (*P =* 0.0432, Figure 2a) and +2.4 ± 5.5% higher for 60Post (*P =* 0.0148, Figure 2a). Between conditions, at 60 min post‐meal FMD was +2.4 ± 4.3% higher for IP (*P =* 0.0401, Figure 2a) and +1.6 ± 4.1% higher for 60Post (*P =* 0.0136, Figure 2a), compared to 30Pre. At 120 min post‐meal, FMD was +2.0 ± 3.7% higher for IP (*P =* 0.0361, Figure 2a) compared to Control. There was no difference over time or between the conditions for FMD AUC (*P =* 0.227, Figure 2b).

**TABLE 1:** Demographics of participants who completed the study.

Demographic	Value (*n* = 24)
**Age, mean ± SD (years)**	29 ± 4
**Sex (M: F)**	12:12
**Height**, mean ± SD **(cm)**	170 ± 11
**Weight**, mean ± SD **(kg)**	74 ± 16
**Body mass index**, mean ± SD **(kg/m^2^)**	25 ± 4
**Lean mass**, mean ± SD **(%)**	69 ± 5
**Fat mass**, mean ± SD **(%)**	27 ± 5
**Strength characteristics**, 1RM (kg)	
**Lunges**	39.30
**Shoulder press (bicep curl for injuries)**	27.08
**Squats**	65.06
**Bench press**	39.44
**Leg‐press**	12.63
**Latissimus dorsi pull down**	9.58

RM = repetiton maximum.

### 3.2 Postprandial glucose

**FIGURE 3:**
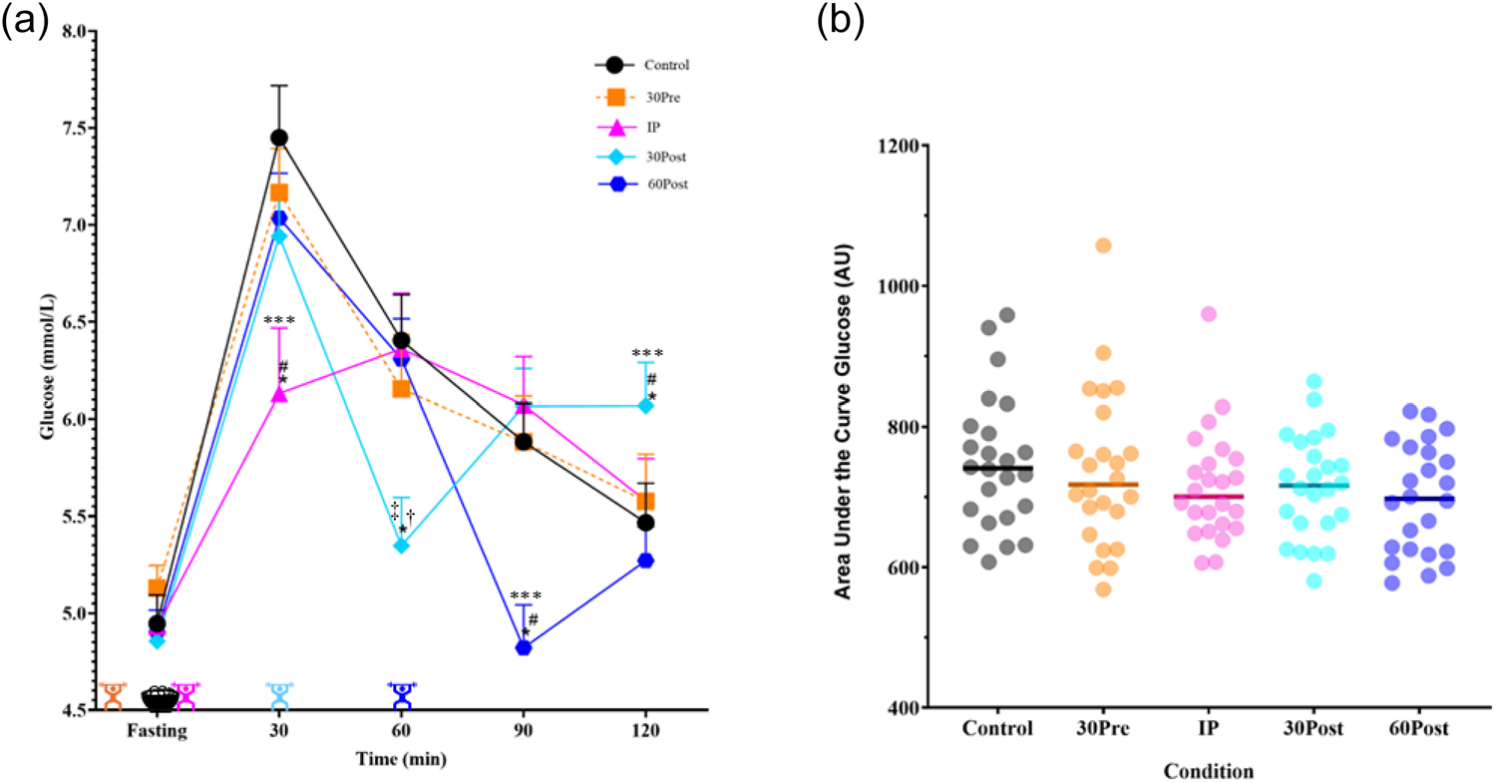
(a) Fasting glucose followed by control, or exercise 30Pre, IP, 30Post and 60Post meal with capillary glucose measured at 30, 60, 90, and 120 min post‐meal (*n* = 24). 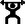 indicates time exercise occurred; 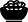 indicates when the meal was eaten. Time point *P < 0.05 compared to Control, *P <* 0.05 compared to pre meal exercise; time point ***P < 0.05 compared to all other conditions and #*P <* 0.05 compared to 30Pre; time point †*P <* 0.05 compared to IP, ‡*P <* 0.05 compared to 60Post exercise. (b) Area under the curve (arbitrary units (AU)), for postprandial capillary glucose with exercise 30 min pre‐, immediately post‐, 30 min post‐ and 60 min post‐meal compared to the no exercise Control (*n* = 24).

**FIGURE 4:**
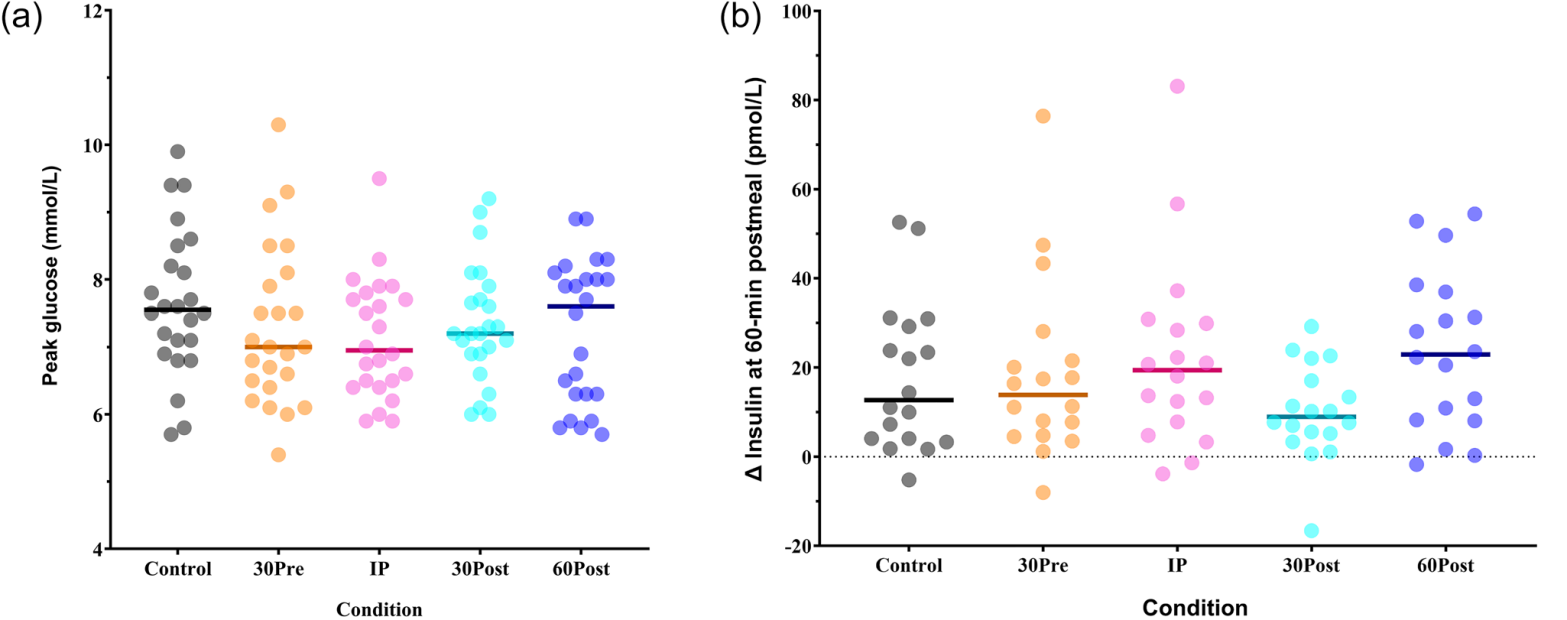
(a) Peak glucose of each participant (dots) with exercise 30 min pre‐, immediately post‐, 30 min post‐, and 60 min post‐meal, and no exercise Control (*n* = 24). (b) Plasma insulin change from fasting to 60 min after a high carbohydrate meal comparing exercise 30 min pre‐, immediately post‐, 30 min post‐ and 60 min post‐meal to a no exercise Control (*n* = 24).

A time‐by‐condition interaction was observed for postprandial glucose (*P =* 0.0001, Figure 3a, Supporting information Table S2). At 30 min post‐meal, postprandial glucose was −1.3 ± 0.4 mmol/L lower in the IP condition, compared to the Control (*P =* 0.0132, Figure 3a) and all other exercise conditions (all *P =* 0.00213, Figure 3a). At 60 min post‐meal, postprandial glucose was −1.1 ± 1.2 mmol/L lower in the 30Post condition compared to the Control (*P =* 0.0185, Figure 3a) and lower compared to IP and 60Post (*P =* 0.0269 and *P =* 0.0412 respectively, Figure 3a). At 90 min post‐meal, postprandial glucose was −1.1 ± 2.0 mmol/L lower in the 60Post condition compared to the Control (*P =* 0.00374, Figure 3a) and lower compared to all other exercise conditions (*P =* 0.0001, Figure 3a). At 120 min post‐meal, postprandial glucose was higher in the 30Post condition by +0.6 ± 0.2 mmol/L compared to the Control (*P =* 0.00925, Figure 3a) and higher compared to all other exercise conditions (*P =* 0.0228, Figure 3a). There was no difference over time or between the conditions for glucose AUC (*P =* 0.348, Figure 3b) or peak glucose (*P =* 0.241, Figure 4a).

### 3.3 Additional cardiometabolic findings

**FIGURE 5:**
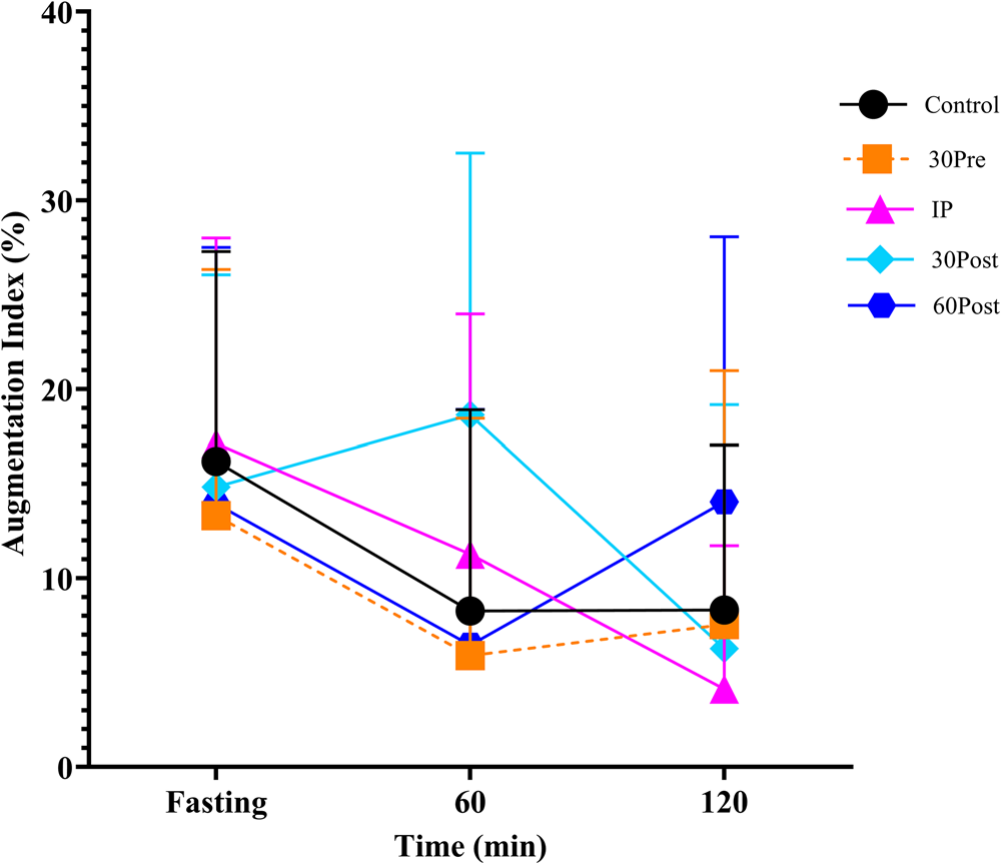
Augmentation Index from fasting to 60 and 120 min post‐meal with exercise 30 min pre‐, immediately post‐, 30 min post‐, and 60 min post‐meal, and no exercise Control (*n* = 24).

A significant time‐by‐condition interaction was observed for AI and systolic blood pressure (*P =* 0.0001 and *P =* 0.0102, respectively, Table 2). However, *post hoc* analysis showed no significant differences between conditions and Control for AI and systolic blood pressure (Figure 5). There were no time‐by‐condition interactions for PWV (*P =* 0.326, Table 2), diastolic blood pressure (*P =* 0.177, Table 2), insulin (*P =* 0.501, Figure 4b), triglycerides (*P =* 0.100), low‐density lipoprotein (LDL) (*P =* 0.172) and high‐density lipoprotein (*P =* 0.0819). Data are provided in Supporting information Table S3.

**TABLE 2:** Vascular measurements of augmentation index (AI) and arterial stiffness via pulse wave velocity (PWV) and blood pressure collected at fasted, 60 and 120 min after a standardized high carbohydrate meal for each condition (*n* = 24).

Variable	Fasting	60 min	120 min	*P*
Augmentation index (units)				**<0.0001**
Control	16.2 ± 11.1	8.3 ± 10.6	8.3 ± 8.7	—
30Pre	13.3 ± 13.0	5.9 ± 12.6	8.3 ± 8.7	0.524
IP	17.1 ± 10.9	11.3 ± 12.7	7.5 ± 13.4	0.981
30Post	14.8 ± 11.2	18.6 ± 13.9	4.1 ± 7.5	0.459
60Post	14.0 ± 13.6	6.5 ± 12.5	6.3 ± 12.9	0.859
PWV (m/s)				**0.325**
Control	5.97 ± 0.88	5.91 ± 0.91	5.73 ± 0.83	—
30Pre	6.00 ± 1.24	5.82 ± 1.29	5.78 ± 1.17	—
IP	5.81 ± 0.63	5.87 ± 0.89	5.75 ± 0.75	—
30Post	5.95 ± 0.67	6.11 ± 0.78	5.88 ± 0.88	—
60Post	5.95 ± 0.68	5.72 ± 0.72	5.83 ± 0.83	—
PWV AUC				**0.611**
Control	706 ± 96			—
30Pre	702 ± 195			—
IP	699 ± 89			—
30Post	722 ± 88			—
60Post	697 ± 89			—
Systolic blood pressure (mmHg)				**0.0102**
Control	105 ± 14	102 ± 9	104 ± 11	—
30Pre	104 ± 9	102 ± 9	103 ± 10	0.928
IP	105 ± 10	102 ± 9	100 ± 10	0.766
30Post	103 ± 8	106 ± 11	99 ± 10	0.781
60Post	103 ± 12	101 ± 11	100 ± 12	0.512
Diastolic blood pressure (mmHg)				**0.177**
Control	72 ± 11	70 ± 8	72 ± 9	—
30Pre	72 ± 8	71 ± 7	71 ± 8	—
IP	72 ± 9	68 ± 8	70 ± 7	—
30Post	71 ± 7	69 ± 8	70 ± 8	—
60Post	71 ± 10	70 ± 9	67 ± 7	—

*P*‐values shown in bold indicate statistical significance. Control, no exercise; 30Pre, exercise 30 min before meal; IP, exercise immediately after meal; 30Post, exercise 30 min after meal; 60Post, exercise 60 min after meal.

## 4 DISCUSSION

This randomized controlled trial investigated how the timing of resistance exercise relative to a meal affects acute postprandial vascular and glucose outcomes in healthy individuals. A key finding was resistance exercise performed immediately and 60 min after a meal improved postprandial endothelial function compared to both the non‐exercise Control and pre‐meal resistance exercise. Overall, all post‐meal resistance exercise conditions improved postprandial glucose responses at the time point 30 min following the exercise bout, compared to the control condition. The improvements in postprandial vascular responses in our study build on existing research showing that post‐meal resistance exercise is more effective for glucose control than exercising before a meal (Engeroff et al., 2023; Solomon et al., 2020; Teo et al., 2018). Taken together, our findings demonstrate that resistance exercise performed after a meal is the most effective strategy for acutely modulating both metabolic and vascular responses in healthy adults. This supports targeted recommendations for postprandial resistance exercise as a practical approach to preserving cardiometabolic health.

Resistance exercise immediately after eating improves postprandial vascular responses and therefore is a useful strategy for attenuating acute responses. In our investigation, resistance exercise immediately post‐meal improved endothelial function by 2.0 ± 3.7% at 60 min, compared to the Control and pre‐meal resistance exercise. Clinically, a 2% FMD increase is associated with a reduced risk of developing cardiovascular disease complications by 26% (Gao et al., 2022; Qiu et al., 2018). Reducing glucose excursions to preserve vascular function requires the combined actions of contraction and insulin‐mediated GLUT4 translocation to amplify glucose uptake (Lai et al., 2010). Therefore, to effectively mitigate acute vascular dysfunction, early postprandial resistance exercise is essential. The current findings indicate that targeting the immediate postprandial period is a promising strategy for improving acute vascular outcomes and merits further investigated regarding their potential to elicit sustained outcomes.

In previous investigations immediate postprandial resistance exercise was shown to blunt postprandial glucose excursion compared to the Control and pre‐meal resistance exercise in healthy adults (Solomon et al., 2020). In our investigation, immediately post‐meal resistance exercise reduced the peak glucose 30 min later by 1.5 ± 1.8 mmol/L, compared to the Control and pre‐meal resistance exercise. Clinically, a 0.7 mmol/L reduction of mean 24‐h glucose is associated with a reduced risk of developing diabetes related complications by 9%, according to the Australian Diabetes Association (2022). Our findings regarding changes in glucose concentrations following immediate post‐meal exercise align with a recent systematic review by Engeroff et al. (2023), which identified resistance exercise timing as a significant moderator showing that the closer exercise occurred to meal ingestion, the greater the reduction in postprandial glucose levels (Engeroff et al., 2023). Together, these findings reinforce the effectiveness of immediate postprandial resistance exercise in lowering post‐meal glucose levels and potentially reducing the risk of diabetes‐related complications. Additional measures of vascular and metabolic function (arterial stiffness, blood pressure, insulin, lipids) were largely unaffected by the meal challenge or resistance exercise in our sample of adults free of cardiometabolic disease.

Pre‐prandial resistance exercise, which initiates GLUT4 translocation prior to meal ingestion (Zouhal et al., 2020), has repeatedly been shown to have minimal lasting effects of postprandial cardiometabolic responses (Slebe et al., 2024), with the exception of high intensity interval exercise (Francois et al., 2014). Consistent with previous literature, this investigation showed pre‐prandial resistance exercise did not differ significantly from the Control in any measured outcome and was significantly less effective than postprandial resistance exercise conditions. It is likely that pre‐meal exercise does not stimulate both contraction‐ and insulin‐mediated glucose uptake to the same extent as immediate postprandial resistance exercise. Therefore, pre‐prandial resistance exercise appears ineffective at attenuating postprandial cardiometabolic outcomes; where possible and if well tolerated, postprandial exercise should be recommended.

### 4.1 Study strengths and limitations

A key strength of this study is the use of a whole‐food meal challenge, rather than a liquid meal or glucose tolerance drink. The carbohydrate load was standardized to individuals’ lean mass, a more physiologically relevant metric as the largest component of lean mass is responsible for approximately 80% of postprandial glucose disposal (Merz & Thurmond, 2020). Exercise intensity was individualized 30% of one‐repetition maximum and controlled for all trials ensuring consistent relative effort across participants.

In our initial protocol we proposed collecting additional PWV/PWA at a 90‐min post‐meal time point; however, logistical constraints prevented the accurate capture of 90 min for the 60Post condition (which had resistance exercise running right up to 90 min), and thus only finger prick glucose data are reported for this time point. A limitation of this study was the presence of missing data for FMD, as some participant files lacked sufficient clarity for accurate analysis. As a result, complete data sets were available for only 19 participants, which is below the target sample size determined in our original power calculation. However, a *post hoc* power analysis indicated that with 18 participants, the study retained 90% statistical power, suggesting that the findings remain robust despite the reduced sample size. Capillary glucose was assessed via finger prick at 30‐min intervals, potentially missing the peak glucose for some participants (if it occurred at 15 min). Additionally, some participants reported mild gastric discomfort with immediate post‐meal resistance exercise, despite all completing the protocol, and no formal survey was provided to evaluate gastrointestinal tolerance. The menstrual cycle phase was not controlled for due to limited or no variability in vascular measurements across the menstrual cycle (Shenouda et al., 2018; Stanhewicz & Wong, 2020; Stanhewicz et al., 2018). Selecting a specific phase of the menstrual cycle (e.g. follicular or luteal) limits the generalisability of findings to only that phase, which represents approximately 20% of the overall cycle (Stanhewicz & Wong, 2020). Accordingly, this study did not manipulate testing around the menstrual cycle, thereby increasing the external validity of the findings, that is, their relevance and applicability to everyday life and typical physiological variation.

### 4.2 Future directions

Long‐term interventions are needed to assess whether daily postprandial exercise leads to sustained improvements in longer‐term clinical markers, such as HbA1c and lipid profiles. Immediate post‐meal exercise was shown to improve both glucose and vascular outcomes. While this presents a simple and actionable message, ‘eat, then go’ rather than waiting a specific period post‐meal, further research is needed to explore the practicalities of implementing this approach in daily life. Future studies should investigate gastrointestinal (GI) responses and tolerability to exercise performed immediately after eating, to better inform personalised and sustainable recommendations. While post‐meal exercise has been found to improve cardiometabolic outcomes in people with and without cardiometabolic disease, it remains unclear whether post‐meal resistance exercise can serve as a prevention strategy for diabetes and cardiovascular disease. Finally, muscle biopsy analysis would provide valuable insight to cellular mechanisms underpinning vascular and glucose outcomes (Pereira & Lancha, 2004).

### 4.3 Conclusion

In conclusion, exercising as soon as possible after a high carbohydrate meal improves endothelial function and attenuates glucose excursions and healthy adults. Given that reducing glucose excursions and increasing vascular distensibility lowers the risk of developing chronic cardiometabolic disease, future research should evaluate the practicality and long‐term effectiveness of integrating early postprandial general exercise in daily living for the prevention of cardiometabolic disease.

## AUTHOR CONTRIBUTIONS

Laura K. Smith and Monique E. Francois: conception and design; Laura K. Smith, Lauren Roach, Courtney R. Chang, Kate Oetsch, Evelyn B. Parr, and Monique E. Francois performed experiments; Laura K. Smith and Monique E. Francois analysed data; Laura K. Smith and Monique E. Francois interpreted results of experiments; Laura K. Smith prepared figures; Laura K. Smith drafted manuscript; Laura K. Smith, Evelyn B. Parr, and Monique E. Franocis edited and revised manuscript. All authors have read and approved the final version of this manuscript and agree to be accountable for all aspects of the work in ensuring that questions related to the accuracy or integrity of any part of the work are appropriately investigated and resolved. All persons designated as authors qualify for authorship, and all those who qualify for authorship are listed.

## CONFLICT OF INTEREST

None declared.

## Supporting information




**Supplementary Figure S1**. CONSORT diagram. Flowchart of participant assessment and subsequent data analysis through the study.

**Supplementary Table S1**. %FMD data for each of the time points for the resistance exercise and the no exercise Control (*n* = 19).
**Supplementary Table S2**. Blood glucose data for each of the time points for the resistance exercise conditions and the no exercise Control (*n* = 24).
**Supplementary Table S3**. Blood lipids and insulin data collected at fasted and 60 min after a standardized high carbohydrate meal for each condition (*n* = 24).

## Data Availability

The protocol as well as datasets generated and/or analysed for this review are available from the corresponding author upon reasonable request.
