# Supplementary material for: Report results: Impact of whole‐body resistance exercise timing on mitigating hyperglycaemia‐induced vascular dysfunction in healthy adults
Source: Exp Physiol. 2026 Mar 9;111(4):1657–68. doi: 10.1113/EP093099 (PMC13140654; doi:10.1113/EP093099)
Supplement: Supplementary file 1 — Supplementary Figure S1. CONSORT diagram. Flowchart of participant assessment and subsequent data analysis through the study. [file EPH-111-1657-s002.pdf]

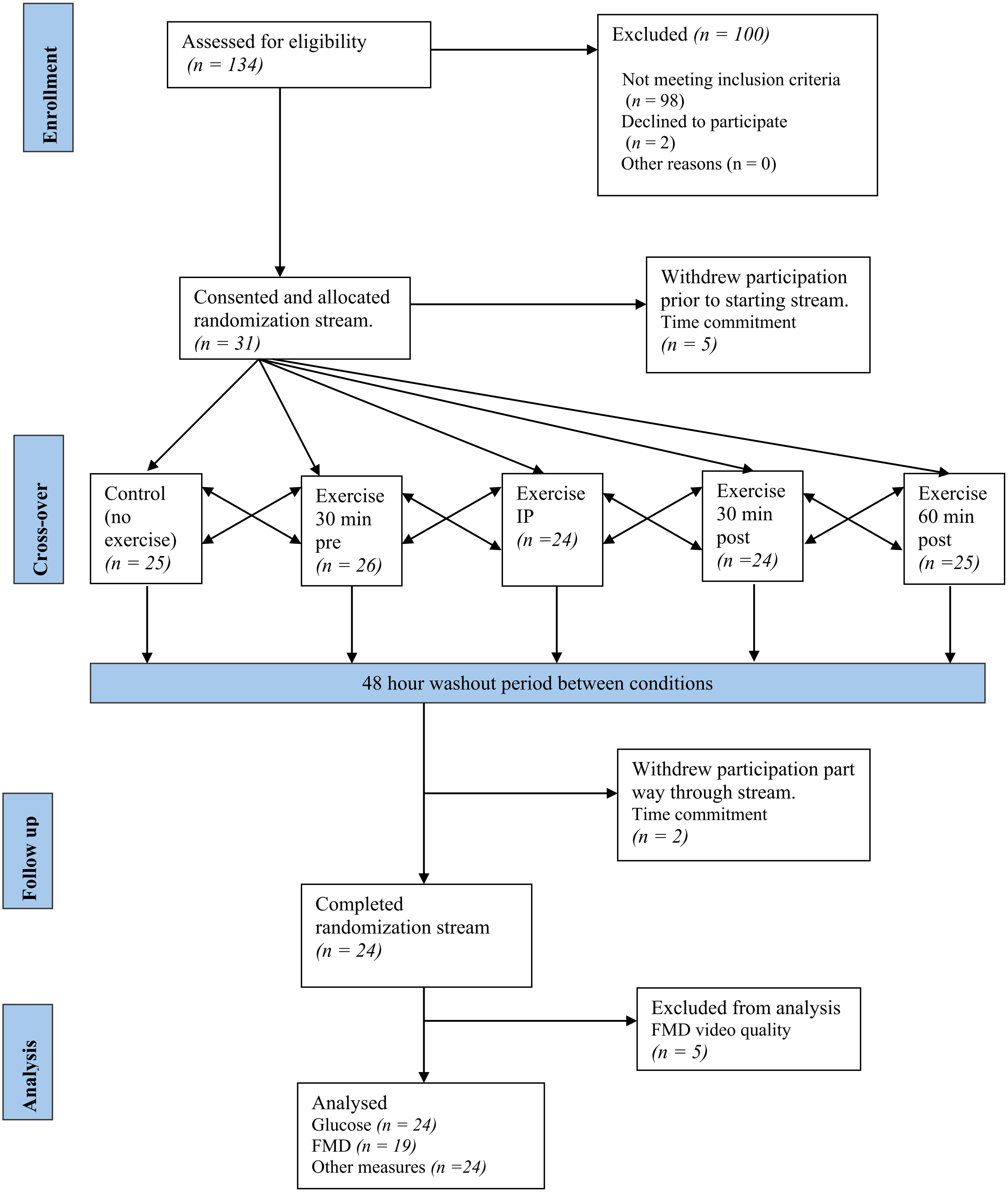

Supplementary Figure 1. CONSORT diagram. Flowchart of participant assessment and subsequent data analysis through the study.
