# Supplementary material for: Report results: Impact of whole‐body resistance exercise timing on mitigating hyperglycaemia‐induced vascular dysfunction in healthy adults
Source: Exp Physiol. 2026 Mar 9;111(4):1657–68. doi: 10.1113/EP093099 (PMC13140654; doi:10.1113/EP093099)
Supplement: Supplementary file 2 — Supplementary Table S1. %FMD data for each of the time points for the resistance exercise and the no exercise Control (n = 19). Supplementary Table S2. Blood glucose data for each of the time points for the resistance exercise conditions and the no exercise Control (n = 24). Supplementary Table S3. Blood lipids and insulin data collected at fasted and 60 min after a standardized high carbohydrate meal for each condition (n = 24). [file EPH-111-1657-s001.docx]

Supplementary Figure and Tables

**Supplementary Figure 1**. CONSORT diagram. Flowchart of participant assessment and subsequent data analysis through the study.

**Supplementary Table 1.** %FMD data for each of the time points for the resistance exercise and the no exercise Control (n=19).

| Condition | 0 | 60 | 120 |
| --- | --- | --- | --- |
| Control | 6.50 ± 1.94 | 6.04 ± 3.10 | 5.82 ± 2.35 |
| 30Pre | 6.26± 1.45 | 5.51±2.01 | 7.38±3.34 |
| IP | 5.88±1.57 | 8.20±4.05*# | 7.76±3.10^*^ |
| 30Post | 5.74 ±1.34 | 7.33±4.34 | 7.32±3.02 |
| 60Post | 5.92±1.56 | 8.91±5.28*^#^ | 6.22±3.34 |

*Significant Post-hoc test condition compared to Control. ^#^ Significant Post-hoc test compared to 30-pre.

**Supplementary Table 2.** Blood glucose data for each of the time points for the resistance exercise conditions and the no exercise Control (n=24).

| Condition | 0 | 30 | 60 | 90 | 120 |
| --- | --- | --- | --- | --- | --- |
| Control | 4.94 ± 0.72 | 7.45 ± 1.31 | 6.40 ± 1.15 | 5.88 ± 0.96 | 5.46 ± 0.99 |
| 30Pre | 5.12 ± 0.57 | 7.16 ± 1.11 | 6.15 ± 1.36 | 5.88 ± 1.15 | 5.57 ± 1.18 |
| IP | 4.93 ± 0.53 | 6.13 ± 1.34*#*** | 6.36 ± 1.05 | 6.07 ± 0.89 | 5.57 ± 0.90 |
| 30Post | 4.85 ± 0.48 | 6.94 ± 1.08 | 5.34 ± 1.21*& | 6.06 ± 0.94 | 6.06 ± 1.08*#*** |
| 60Post | 4.90 ± 0.55 | 7.03 ± 1.12 | 6.30 ± 1.01 | 4.82 ± 1.08*#*** | 5.27 ± 0.85 |

*Timepoint P<0.05 compared to Control, P<0.05 compared to pre meal exercise, ***Timepoint P<0.05 compared to all other conditions and #Timepoint P<0.05 compared to 30Pre, +Timepoint P<0.05 compared to IP, ^&^Timepoint P<0.05 compared to 60Post exercise

**Supplementary Table 3.** Blood lipids and insulin data collected at fasted and 60 after a standardized high carbohydrate meal for each condition (n=24).

| Variable | Condition | | Fasting | | 60 | | P value |
| --- | --- | --- | --- | --- | --- | --- | --- |
| *HDL (mmol/L)* | Control | 1.42 ± 0.32 | | 1.37 ± 0.36 | | 0.0819 | |
|  | 30Pre | 1.44 ± 0.37 | | 1.42 ± 0.34 | | - | |
|  | IP | 1.89 ± 1.65 | | 1.40 ± 0.32 | | - | |
|  | 30Post | 1.42 ± 0.35 | | 1.48 ± 0.31 | | - | |
|  | 60Post | 1.44 ± 0.33 | | 1.45 ± 0.32 | | - | |
| *LDL (mmol/L)* | Control | 2.29 ± 0.70 | | 2.76 ± 0.76 | | 0.172 | |
|  | 30Pre | 2.29 ± 0.74 | | 2.70 ± 0.72 | | - | |
|  | IP | 2.65 ± 1.29 | | 2.85 ± 2.61 | | - | |
|  | 30Post | 2.42 ± 0.68 | | 3.01 ± 0.93 | | - | |
|  | 60Post | 2.34 ± 0.69 | | 2.82 ± 0.78 | | - | |
| *Triglycerides (mmol/L)* | Control | 1.00 ± 0.52 | | 1.05 ± 0.79 | | 0.100 | |
|  | 30Pre | 1.20 ± 1.25 | | 0.97 ± 0.43 | | - | |
|  | IP | 1.48 ± 1.52 | | 0.99 ± 0.48 | | - | |
|  | 30Post | 0.87 ± 0.31 | | 1.05 ± 0.49 | | - | |
|  | 60Post | 0.89 ± 0.32 | | 0.99 ± 0.49 | | - | |
| *Insulin (mmol/L)* | Control | 3.15 ± 1.91 | | 20.74 ± 17.68 | | 0.501 | |
|  | 30Pre | 3.92 ± 2.69 | | 22.40 ± 20.94 | | - | |
|  | IP | 3.60 ± 2.91 | | 25.73 ± 22.14 | | - | |
|  | 30Post | 4.32 ± 5.23 | | 14.40 ± 8.82 | | - | |
|  | 60Post | 4.08 ± 3.56 | | 27.91 ± 18.27 | | - | |
